# Microfluidic fabrication of pectin-coated liposomes for drug delivery

**DOI:** 10.1007/s13346-025-01812-0

**Published:** 2025-02-22

**Authors:** Anitta Lutta, Qian Liu, Gabriel Kristian Pedersen, Mingdong Dong, Holger Grohganz, Line Hagner Nielsen, Signe Tandrup Schmidt

**Affiliations:** 1 Department of Infectious Disease Immunology, Statens Serum Institut, Artillerivej 5, 2300 Copenhagen, Denmark; 2 Department of Health Technology, Technical University of Denmark, Ørsteds Plads 345C, 2800 Kgs. Lyngby, Denmark; 3 Interdisciplinary Nanoscience Center, Aarhus University, Gustav Wieds Vej 14, 8000 Aarhus, Denmark; 4 Department of Pharmacy, University of Copenhagen, Universitetsparken 2, 2100 Copenhagen, Denmark

**Keywords:** Cationic adjuvant formulation, Biopolymers, Nanoparticles, Electrostatic adsorption, Polymer-coating

## Abstract

Polymer coating of nanoparticulate drug delivery systems may enhance the efficacy of oral delivery. Cationic liposomes were coated with pectin biopolymers using microfluidics, with systematic variation of process parameters to optimize pectin-coated liposome fabrication. A pectin/liposome weight ratio of 0.7 and a microfluidic flow rate ratio of 2:1 pectin:liposome were found to be optimal. The resulting formulations displayed particle sizes at least threefold the size of uncoated liposomes, while the surface charge shifted to a highly negative value, indicating full pectin coating of the particles. Further microscopic characterization of the pectin-coated liposomes revealed that the pectins formed a polymeric network within which the liposomes were dispersed or attached. Stability studies revealed that pectin-coated liposomes remained stable during storage, with no displacement of the coating. We determined that microfluidics is a robust method for preparing pectin-coated liposomes, despite the structural differences between the pectins, geometry of the microchip used, and pectin/liposome concentration. Ultimately, the use of microfluidics in formulation development could be highly beneficial, as the process parameters can be easily modified and the process is easily scalable and inexpensive. Additionally, pectins can offer protective properties to the liposomes particularly during oral drug delivery.

**Graphical Abstract:**

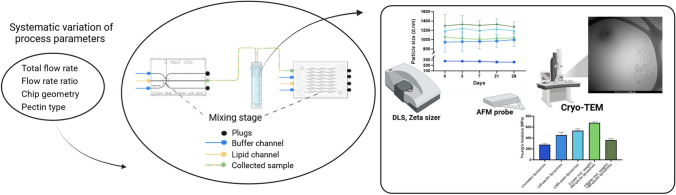

## Introduction

Liposomes are among the most commonly researched nanocarriers for drug delivery because of their versatility, biocompatibility, and biodegradability [1]. They lack site specificity; therefore, they can be easily manipulated for specific use in the drug delivery field [2]. For example, stimuli-responsive elements can be attached to liposomes and polymeric coatings, and hybrid liposomes can be prepared by surface modification of liposomes [3–5]. Particularly in drug delivery, polymeric coatings can be applied to liposomes to tune the mucoadhesive properties of particles. Altering mucoadhesive properties can result in higher retention of drug particles at the site of uptake, resulting in a higher degree of drug absorption [3, 6].

Currently, research is focused on implementing more environmentally sustainable processes, including the use of biopolymers such as chitosan, hyaluronic acid, and pectins [7]. Pectins are biocompatible, biodegradable, generally regarded as safe (GRAS) [8], and have a track record in the pharmaceutical industry. For instance, pectin has been established as a binding agent in tablets, a gelling agent in gel beads for sustained drug delivery, and a bio-adhesive in patches [9]. Pectin is an anionic polysaccharide that consists mainly of repeating galacturonic acid monomers with different side chains, resulting in polymeric properties [10]. The pectin side chains can also be manipulated to vary the degree of methyl esterification on the galacturonic acid units [10, 11]. These alterations result in different pectins, namely high-methoxylated (HM) pectins with a degree of esterification (DE) > 50% or low-methoxylated (LM) pectins with a DE < 50%. Furthermore, LM pectins can be amidated, resulting in low-methoxylated amidated (LMA) pectins [12]. These structural differences give pectins a unique profile for mucoadhesion and mucopenetration, critical for increasing liposome proximity to intestinal epithelial cells during oral drug delivery [13, 14]. Additionally, pectins can enhance liposome in vivo stability after oral delivery by protecting against premature degradation in the harsh gastrointestinal environment [15].

Polymeric coatings of liposomes can be performed using different approaches that have been extensively studied [3, 15–18]. Conventionally, polymers are chemically conjugated to the surface of the bilayer membrane of liposomes. This process involves covalent bonding between functional groups on the polymer and reactive groups on the surface of the liposomes [17]. Conjugation of polymers onto the surface of liposomes can be achieved by solvent evaporation [18] or polymerization [19]. These methods are often not ideal for the development of novel delivery systems because they are time-consuming, rely on organic solvents, and are difficult to scale up. Alternatively, polymers can be deposited on the surface of liposomes in a non-covalent manner by relying on hydrogen bonds [9], as well as electrostatic attraction. Oppositely charged polymers can form strong electrostatic attractions with the liposome surface, leading to stable polymeric coatings [3, 15, 16]. Such non-covalent methods of polymer coating can be achieved by various methods, for example, mixing using a peristaltic pump or microfluidics. Microfluidics has been established for the preparation of self-assembled drug delivery systems by interfacing multiple streams of several reagents [20]. It is a highly reproducible method that allows for precise control over the mixing process [21] and is therefore suitable for high-throughput production. However, the use of microfluidics in the pectin coating of liposomes remains an understudied area, and more research in this field is relevant in developing robust drug delivery systems.

In this study, we used microfluidics to produce pectin-coated liposomes. We demonstrated that microfluidics is a robust and reproducible method for coating cationic particles with structurally different pectins. We systemically studied critical process parameters such as the polymer concentration, flow rate ratio between the polymer and liposomes, and geometry of the microchip used in the fabrication process. The goal was to define an optimal and reproducible fabrication process for pectin-coated liposomes that resulted in uniform particles that remained stable during storage and had a desirable particle size.

## Materials and Methods

### Materials

Cationic adjuvant formulations (CAF^®^) are cationic liposomes used as adjuvants for vaccine delivery [22]. CAF04 liposomes were used to prepare pectin-coated liposomes and are based on two lipids, N,N-dimethyl-N,N-dioctadecylammonium bromide (DDA) and glycerolipid monomycoloyl glycerol (MMG) both purchased from Niels Clauson-Kaas A/S (Farum, Denmark). Pectins with varying structural properties (Table 1) were kindly provided by CP Kelco ApS (Lille Skensved, Denmark). All other reagents were of analytical grade.

**Table 1:** Pectins’ degree of esterification (%D.E) and amidation (%D.A) and molecular weights

	% D.E	% D.A	Mol. Weight (kDa)
LM-pectin liposomes	31	-	113
LMA-pectin liposomes	29	18	139
(Lower mol. weight) HM-pectin liposomes	71	-	164
(Higher mol. weight) HM-pectin liposomes	71	-	243

### Liposome preparation

In initial optimization studies, CAF04 liposomes were prepared using microfluidics (Dolomite Microfluidics, UK). This entailed dissolving DDA and MMG lipid powders in ethanol followed by mixing with acetate buffer (5 mM, pH 4.5), as described previously [23, 24]. Briefly, the lipids in ethanol and aqueous acetate buffer were mixed in a 5-way input chip (with 2 inlets plugged off) at FRR 15:1 to obtain a final liposome concentration was 2.5:0.5 mg/ml DDA:MMG. Preparation of liposomes via the microfluidic method resulted in the liposomes having 6.25% residual ethanol, therefore for subsequent studies, liposomes were prepared using the conventional thin-film hydration technique, followed by high-shear mixing (HSM). Briefly, DDA and MMG lipid powders were dissolved in ethanol (99%) at a 5:1 weight ratio. The ethanol was subsequently evaporated, resulting in dry lipid powder films, which were flushed with nitrogen gas to remove trace amounts of ethanol. The lipid powders were rehydrated in Milli-Q water at 60 °C using a Heidolph Silent Crusher equipped with a 6F shearing tool (Heidolph Instruments GmbH, Schabach, Germany) at 26,000 rpm. The final liposome formulation had a lipid concentration of 3.75:0.75 mg/ml DDA:MMG.

### Pectin coating of liposomes

**Fig. 1:**
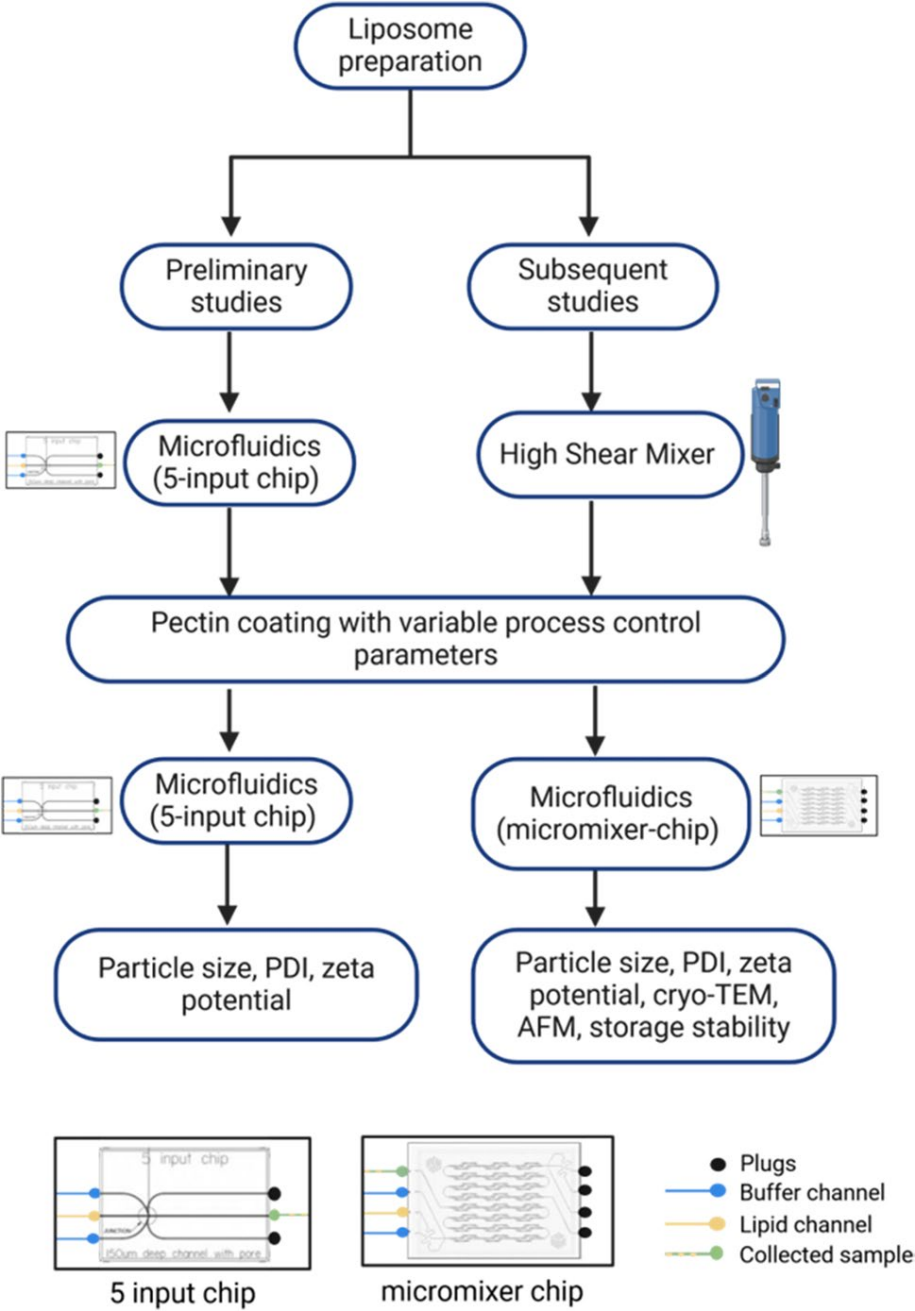
Schematic workflow of liposome preparation and pectin coating as well as characterization

Pectin stock solutions were prepared by dissolving 1% weight of pectins in 5 mM sodium acetate buffer at pH 4.5. The stock solutions were further diluted to a working concentration of 0.1% or 0.15% by weight using 5 mM sodium acetate buffer at pH 4.5. Subsequently, pectin coating of the liposomes was carried out using a droplet microfluidic system (Dolomite Microfluidics, Royston, United Kingdom) based on laminar flow. Pectin solutions were applied as the continuous phase at either 1 mg/mL or 1.5 mg/mL, while the CAF04 liposomes were dispersed into the pectin solution. The flow rate ratios (FRR) varied between 2:1, 3:1, 4:1, and 5:1 pectin:liposomes resulting in total flow rate (TFR) was varied from to 3–6 mL. A hydrophilic 5-way input chip (with 2 inlets plugged off) was used in the initial optimization studies, while the micromixer chip (with 4 inlets plugged off) used for the subsequent studies, as shown in Fig. 1. The micromixer chip having more mixing channels due to the herringbone structure, was used to enhance mixing of pectins with liposomes. The microchips were fitted on a H interface and a Linear 4-way Connector, which was connected by FEP tubing connections with outer diameter (OD) and inner diameter (ID) (OD 1.6 mm and ID 0.25 and 0.8 mm respectively) to a Mitos p-pump basic. A Mitos Flow Rate Sensor (flow rate limit 0.2–5 mL) was also connected in-line between the pump and the microfluidic device to control the flow rate. A Mitos compressor was used to supply the chambers with pressure, and a Mitos microscope was used to observe the flow in the microchips. Dolomite flow control center software (version A.6) was used to set the pressure and flow rates as well as to monitor the changes in flow rates during fabrication. At FRR 2:1, the resulting pectin/liposome ratio was 0.7, whereas at FRR 3:1, the resulting ratio was 1. At an FRR of 4:1, the resulting pectin/liposome ratio was 1.3, whereas that of FRR 5:1 was 1.7.

### Particle size and surface charge characterization of pectin coated liposomes

The mean particle size distribution (Z-average), polydispersity index (PDI), and zeta potential of the pectin-coated liposomes were determined by DLS using a Zetasizer Nano ZS (Malvern Instruments, Worcestershire, UK) fitted with a 633 nm laser and 173° detection optics. Malvern DTS v.6.20 software (Malvern Instruments) was used for data acquisition and analysis. The measurements were repeated three times for each sample. The values for pure water were used to determine the viscosity and refractive index. A Nanosphere™ Size Standard (220 ± 6 nm, Duke Scientific Palo Alto, CA, USA) was used to verify the performance of the instrument. Uncoated liposomes were used as controls for the measurements. Pectin-coated particles were stored at 4 °C for up to 28 days, and their colloidal stability of the pectin coated particles was analyzed by DLS at intervals.

### Morphology of pectin-coated liposomes

Pectin-coated liposomes were imaged using cryogenic transmission electron microscopy (cryo-TEM). Three microliters of the sample were applied to a hydrophilized lacey carbon 300 mesh copper grid (Ted Pella Inc., California, USA). The excess sample on the grid was blotted with filter paper at a blotting time of 5 s, blotting force of 0, temperature of 4⁰C, and 100% humidity (FEI Vitrobot IV, Eindhoven, The Netherlands), and was rapidly plunged into liquid-nitrogen cooled ethane (−180⁰C). Sample observations were performed using a Tecnai G2 20 transmission electron microscope (FEI, Eindhoven, Netherlands) at a voltage of 200 kV under a low dose rate. Images were recorded with an FEI Eagle camera 4kx4k at a nominal magnification of 29 kX.

Atomic force microscopy (AFM) was used to confirm the morphology of the pectin-coated particles. A 10 μL sample was drop-cast onto freshly cleaved mica. After 5 min of incubation, the excess liquid was removed, rinsed, and the mica substrate was allowed to air-dry at room temperature. Peak Force Tapping^®^ (Bruker, Corporation, Massachusetts USA) in air mode was applied for imaging by Multimode^®^ VII AFM (Bruker, Corporation, Massachusetts USA). RFESP-75 silicon cantilevers (Bruker AFM probes) with a spring constant of 1.98 N/m and tip radius of 8 nm were used for imaging, and the setpoint was less than 5 nN. Images were collected with a resolution of 256 × 256 pixels and a scan rate of 1 Hz. Data analysis, including the flattening of raw images for the morphology and calculation of Young’s modulus was carried out by SPIP™ (Scanning Probe Image Processor) software package (Image Metrology, Lyngby, Denmark).

### Statistics

Ordinary one-way ANOVA with Tukey’s multiple comparisons test was applied to the statistical analysis of the particle size, PDI, zeta potential and Young’s modulus using GraphPad Prism 10.0.2 (2 3 2) (GraphPad Software, Boston, MA, USA).

## Results

### Optimization of pectin-coating process

**Fig. 2:**
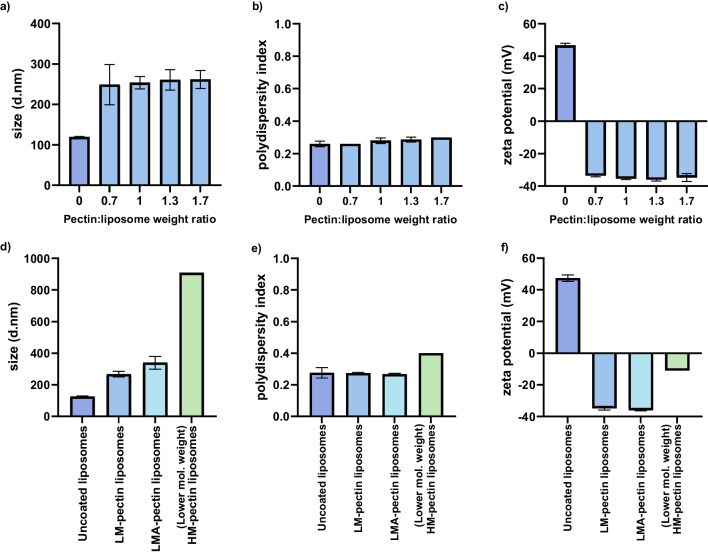
Characterization of pectin coated liposomes after the pilot study. The cationic liposomes were prepared using microfluidics with a lipid concentration of 2.5:0.5 mg/ml DDA:MMG. The starting concentration of pectin was 1 mg/mL and pectin coated liposomes were prepared with a 5-input microchip, with 2 inlets plugged off. **a**,**b** and **c** show the particle size, PDI and zeta potential characterization. a)-c) particles prepared with LM-pectin, and varying pectin/liposome ratio. **d**-**f** prepared with different pectins, *(n* = *3,* ± *SD)* for all except lower mol. weight HM-pectin (*n* = 2)

Changes in particle size and surface charge after coating were used to determine the success of the pectin-coating process. The particle sizes appeared to increase for the coated liposomes compared with the uncoated liposomes (Fig. 2). This increase in particle size, while maintaining a PDI under 0.3 was indicative of the successful adsorption of the pectins on the surface of the liposomes. Moreover, the shift in particle surface charge from cationic to anionic after pectin coating was used to study pectin saturation on liposomes. The pectin/liposome ratio was carefully adjusted to determine the influence of pectin concentration on the particle size and zeta potential. Furthermore, the particle size increased twofold after pectin coating (Fig. 2a) while maintaining the PDI (Fig. 2b). Notably, the cationic liposomes became anionic after microfluidics were mixed with pectins, with no further changes in the zeta potential despite additional pectin (Fig. 2c).

Particles coated with both LM and LMA-pectins showed a threefold increase in size, with a PDI similar to that of the uncoated liposomes, and the zeta potential significantly changed to anionic after pectin coating (Fig. 2d-f). In contrast, particles coated with HM-pectins had a significant increase in particle size (ninefold), with a PDI higher than that of uncoated liposomes (Fig. 2d-e). In addition, the zeta potential, although negative, was closer to neutral than that of the other pectins (Fig. 2f).

### Characterization of size and surface charge of pectin-coated liposomes

Following the pilot studies, we established that limiting the pectin/liposome ratio to 0.7 was optimal. Therefore, to maintain this ratio while increasing the final CAF04 ratio, we increased the concentration of the starting materials, i.e. pectin at 1.5 mg/mL and 3.75:0.75 mg/ml DDA:MMG. This resulted in formulations containing 1 mg/mL pectin and 1.25:0.25 mg/ml DDA:MMG i.e. pectin/liposome ratio 0.7. The particle sizes were studied to characterize the coating process. Pectin-coated liposomes were significantly larger than uncoated liposomes (p = 0.001). However, there were no significant differences in sizes between the pectin groups. Moreover, a PDI of 0.3 was indicative of uniformly dispersed particles across all groups (Table 2). Similar to the results from the pilot studies, particles prepared with LM-pectins and LMA-pectins had stronger negative surface charges compared to HM-pectins (Table 2).

**Table 2:** Characterization of liposomes after pectin coating on day of production (day 0) representative of n = 3 ± SD, with p-values from ordinary one-way ANOVA

Formulations	Sizes (d.nm)	PDI	Zeta potential (mV)
Uncoated liposomes (before microfluidics)	219 ± 12	0.3 ± 0.1	
Uncoated liposomes (after microfluidics)	220 ± 12	0.3 ± 0.1	
LM-pectin liposomes	965 ± 178 *p* < *0.001*	0.3 ± 0.1 p < 0.024	−38 ± 1.74 *p* < *0.001*
LMA-pectin liposomes	1211 ± 106 *p* < *0.001*	0.3 ± 0.1 p < 0.086	−40 ± 3.17 *p* < *0.001*
(Lower mol. weight) HM-pectin liposomes	1098 ± 140 *p* < *0.001*	0.2 ± 0.1 p < 0.972	−18 ± 3.68 *p* < *0.001*
(Higher mol. weight) HM-pectin liposomes	1322 ± 204 *p* < *0.001*	0.3 ± 0.1 p < 0.0265	−14 ± 6.04 *p* < *0.001*

**Fig. 3:**
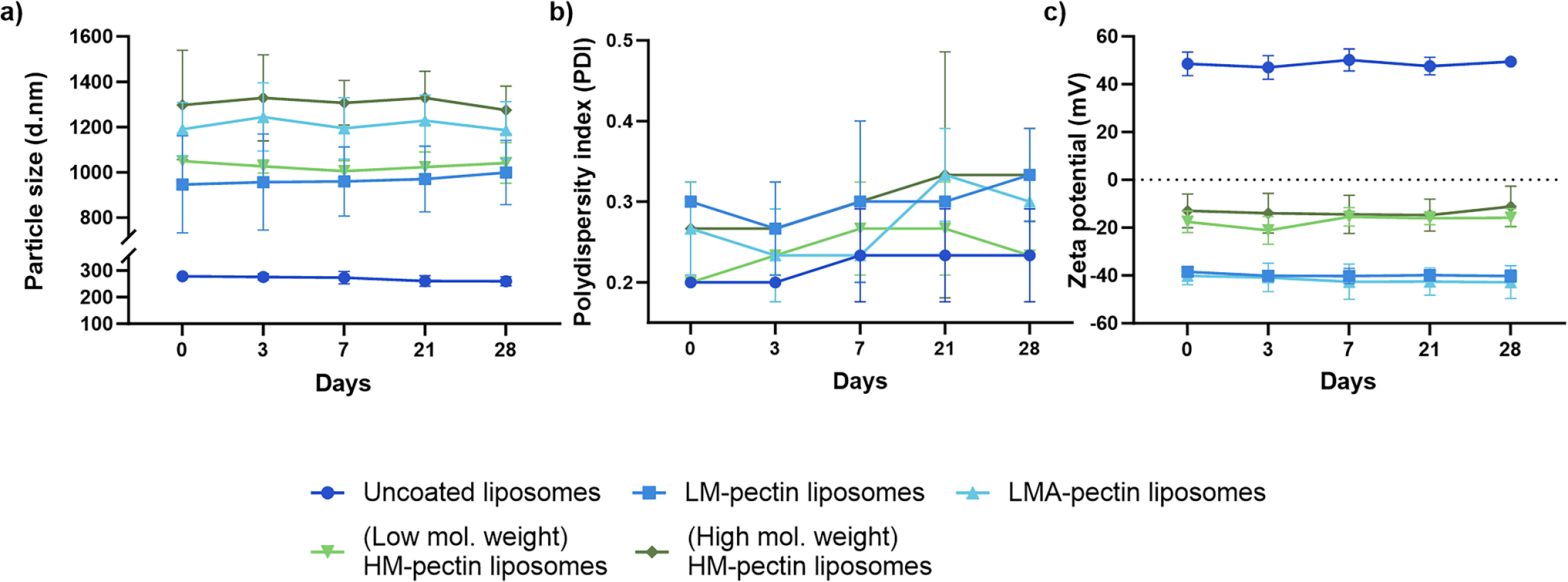
Depicting the changes in size, PDI and surface charge of particles during storage. **a**, **b** and **c** illustrate the size characterization, PDI and zeta potential respectively, *(n* = *3* ± *SD).* The cationic liposomes were prepared using high-shear mixing (HSM), with a lipid concentration of 3.75:0.75 DDA:MMG, the pectin concentration of 1.5 mg/mL was used and a micromixer microchip (with 4 inlets plugged off) used for pectin coating. The final lipid concentration was 1.25:0.25 DDA:MMG, pectin/liposome ratio 0.7. The data from particle characterization on day 0 is also summarized in Table 2

We determined the storage stability of the pectin-coated particles, and the particle sizes and zeta potentials were characterized over time (Fig. 3).

Although there were minimal changes in particle size during storage, these changes were at par with the changes in particles that were uncoated. Additionally, the particle sizes measured during storage (Fig. 3a) were not significantly different from those of freshly prepared particles (day 0, Table 2), indicating that all formulations were colloidally stable within the storage period.

### Morphology of pectin-coated liposomes

**Fig. 4:**
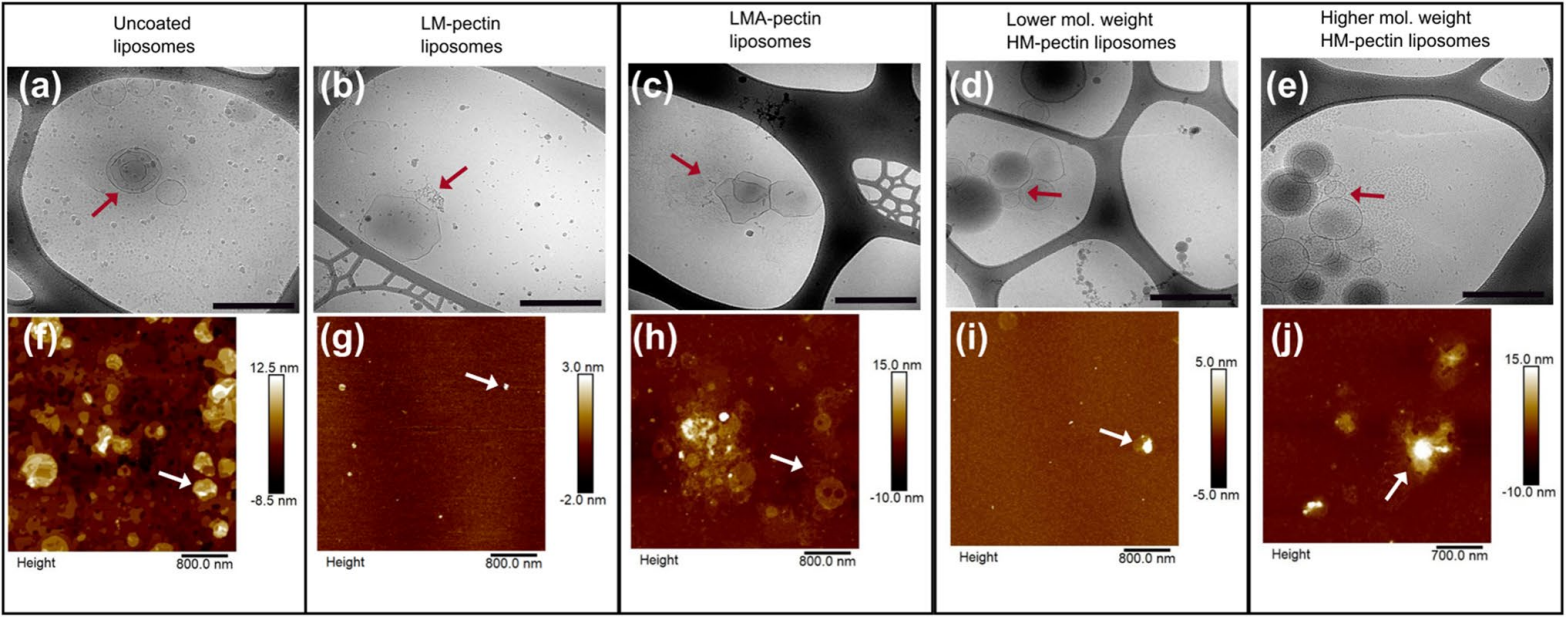
Morphological characterization of pectin-coated liposomes, (**a-e**) show cryo-TEM micrographs with the scale bar indicating 200 nm and (**f-j**) show AFM micrographs. The arrows highlight the presence of liposomes (**a**, **f**) or liposomes attached to the polymeric complex as in (**b-e** and **g-h**)

The cryo-TEM micrographs revealed that the uncoated liposomes contained both uni-lamellar vesicles and multi-lamellar vesicles (Fig. 4a). Additionally, the AFM images revealed contrasting brightness levels among the vesicles, indicating height differences which indicated the presence of multilamellar vesicles (Fig. 4 f-j). Similar to the cryo-TEM micrograph of the uncoated liposomes, (Fig. 4a), the AFM micrographs showed liposomes of varying sizes (Fig. 4b).

After coating, the vesicles appeared to be clustered together, and a pectin network was evident in the micrographs, particularly LM and LMA-pectins (Fig. 4b,c). Furthermore, the pectin chains appeared to be attached to the liposomes rather than surrounding the vesicles, as initially hypothesized. It was also evident that different liposomes were interconnected by the pectin network (Fig. 4b-e). Moreover, cryo-TEM micrographs of the pectin-coated particles also revealed that LM and LMA-pectins formed loops rather than trains (Fig. 4b, c). However, it was unclear whether the loops were formed on the HM-pectin-coated liposomes (Fig. 4d, e). Instead, the vesicles appeared to have darker shadows, as indicated by the arrows (Fig. 4d, e). Nevertheless, the micrographs obtained from AFM further confirmed the formation of loops of pectin chains in HM-pectin-coated liposomes, as well as the presence of a pectin moiety surrounding the liposomes (Fig. 4i, j).

**Fig. 5:**
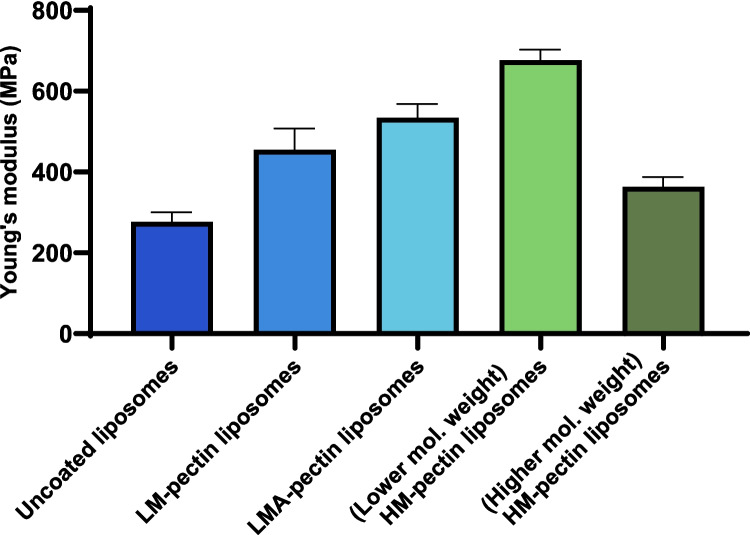
Changes in Young’s modulus calculated after AFM measurements of pectin-coated liposomes as well as uncoated liposomes

Adsorption on liposomes can alter stiffness of liposomes, which can be determined using Young’s modulus calculations [25, 26]. The results from Young’s modulus calculations revealed that the rigidity was higher in pectin coated liposomes, which indicated that the pectin successfully adsorbed on the surface of the liposomes (Fig. 5).

## Discussion

During pilot studies, liposomes were prepared using microfluidics, resulting in smaller particle sizes. However, this process is yet to be optimized to remove residual ethanol. Therefore, subsequent studies were carried out with liposomes prepared using the conventional method, resulting in slightly larger particle sizes, as well as both multi-lamellar and uni-lamellar vesicles. Although the initial particle sizes were slightly larger, we observed a similar trend with liposomes coated with LM pectins having slightly smaller particles compared to the other groups. Even so, the changes in particle size were largest for the higher-molecular-weight HM-pectin-coated liposomes and smallest for LM-pectin-coated liposomes, which is in agreement with earlier findings [27]. This could be due to the molecular weight of the polymers as well as the degree of esterification. The molecular weight of polymers has been previously reported to influence the size and surface charge of polymer-coated liposomes [13]. Pectins with higher molecular weights have been shown to result in larger particle sizes [13, 27]. This is largely due to greater steric hindrance, where the larger adsorbed layer can prevent flat packing of the polymer around the liposomes, leading to both larger particle sizes and zeta potentials closer to neutral compared to LM and LMA-pectin-coated liposomes [13]. Surprisingly, the lower-molecular-weight HM-pectin resulted in smaller particle sizes compared to the higher-molecular-weight HM-pectin. However, the particles prepared with the lower-molecular-weight HM-pectin appeared to be smaller in size than those prepared with LMA-pectin; this difference was not significant. Moreover, the molecular weight of the lower-molecular-weight HM-pectin was closer to that of LMA-pectin than that of the higher-molecular-weight HM-pectin.

Electrostatic interactions primarily drive binding between pectins and cationic liposomes [27]. The -COOH groups on the pectins give pectins a characteristic negative charge. Therefore, when negatively charged pectin surfaces encounter positively charged surfaces on liposomes, strong electrostatic attraction occurs, resulting in pectin coating on the surface of liposomes. Therefore, changes in the zeta potential of the particles after pectin coating provide information on the nature of the coating process. After fully coating the particles, the zeta potential remained steady despite an increase in the pectin/liposome ratio (Fig. 2c). This could be due to the depletion of available electrostatically charged sites on the surface of liposomes for pectin binding. In addition, increasing the pectin/liposome ratio would result in a low liposome concentration, e.g. 0.5 mg/mL in the case of FRR 5:1, which would limit the dosing possibilities for drug delivery. Instead, one way to increase the final liposome concentration was to increase the starting concentrations of both pectin solutions and liposomes. This resulted in a threefold increase in the final liposome concentration.

Particles coated with HM-pectins exhibited zeta potentials closer to neutral than those coated with LM and LMA-pectins. This could be explained by the higher %DE in HM-pectins, where several functional side groups are esterified. Therefore, fewer negatively charged surfaces are available for electrostatic interactions compared to LM-pectins. Thus, the stronger negative charges on the surface of the liposomes coated with LM-pectins were attributed to the %DE of the pectins. Similarly, the %DE determines the surface charge of the particles coated with LMA-pectins, as they have a similar %DE to LM-pectins, with the addition of amide groups on the LMA-pectins. Other factors that influence the characteristics of the polymer layer around liposomes have been previously reported [13, 27]. For example, Klemetsrud et al. reported that HM-pectins with a lower charge density have fewer groups for electrostatic binding with oppositely charged liposomes [13]. Similarly, Nguyen et al. also reported that LM and LMA-pectins exhibited more negatively charged particles than HM-pectins [27]. Moreover, the negative charge in the pectin-coated samples was indeed due to the coating on the liposomes and not due to unbound pectins, which were removed by repeated ultracentrifugation and washing [27].

Neat CAF04 liposomes are cationic, which prevents vesicles from fusing. It is therefore possible that the clustering of the liposomes observed in the cryo-TEM micrographs (Fig. 4 c-e) was facilitated by the pectins, possibly due to cross-linking. The cross-linking of pectin chains has been correlated with the branching of the side chains, as well as the molecular weight [28]. The branching of pectin chains has been further categorized as the formation of trains, loops, or tails [27]. Higher-molecular-weight pectins tend to form loops and tails because of strong hydrophobic interactions with other sections of the pectin chains. We expected HM-pectin-coated liposomes to contain characteristic loop structures, while the LM and LMA-pectin-coated liposomes had train structures with tightly packed pectin chains around the liposomes. It has previously been reported that pectin side chains that form loops instead of trains lead to larger particle sizes [27]. The loops formed by the pectin chains in the coated liposomes could also explain the large particle sizes obtained by DLS. Because the particles are connected through the pectin network, the light scattering of the particles during DLS measurements would not be as accurate as that in solutions with freely dispersed particles.

The increase in Young’s modulus of the pectin coated particles indicated that their rigidity increased after pectin coating, further suggesting successful adsorption on the liposome surface (Fig. 5). This detected increase in rigidity could be attributed to the aforementioned loop or train structures that form in pectin.

Previous studies have used purified pectins with a cut-off molecular weight of 8 kDa [13, 27]. It is plausible that limiting the molecular weight of pectins results in smaller particle sizes. Additionally, these studies used DPPC/DOTAP liposomes, which had fewer cationic charges than CAF04. It is likely that the degree of electrostatic interaction, which is related to the ratio between cationic and anionic charges, was more significant in CAF04 liposomes, which are strongly cationic. Similarly, previous studies prepared pectin solutions in PBS at a pH of 6.5 [13, 27]. Pectin exhibits unique structural properties that vary with changes in buffer pH and composition [29]. At pH 4.5, near the pKa of pectins, carboxylic groups exist in both protonated and deprotonated states, creating an equilibrium between compact and extended structures due to intermediate electrostatic repulsion. At pH 6.5 and above, carboxylic groups are predominantly deprotonated, increasing negative charge along the pectin chain and resulting in strong repulsive forces that cause the chain to extend and form loops. Therefore, dissolving pectins in acetate buffer likely results in solutions with more trains than loops compared to phosphate buffer. However, this contrasts with the cryo-TEM and AFM imaging results, which showed loop structures in pectin-coated liposomes.

The displacement of the pectin coating was indicated by the zeta potential shifting towards neutral, and aggregation during DLS measurements signaled particles “leaking” out of the polymeric network. However, this was not the case across all formulations, as the coated particles retained their anionic status, with virtually no changes in the zeta potential (Fig. 4c). Moreover, slight changes in the particle sizes were at par with the particle sizes on the day of production (Table 2). Therefore, pectin-coated particles prepared via microfluidics remained stable during storage.

In contrast to the 5-input chip, the micromixer chip incorporates multiple mixing stages, enhancing the interaction between pectin and liposomes. To evaluate the impact of these multiple mixing zones on the liposomes, we passed them through the micromixer chip without pectins. Notably, the liposome sizes remained constant, indicating that this additional mixing did not induce further shear stress on the particles (Table 2).

## Conclusion

In conclusion, microfluidics is a robust technique for the preparation of pectin-coated liposomes. Despite the structural differences between pectins, this method was deemed as fast, reproducible, and with minimal batch-to-batch variations. Ultimately, this current work demonstrates that microfluidics is a suitable method for coating of liposomes with pectins, allowing for simple and fast adjustments of the process parameters, which can streamline the formulation development process. Characterization of the pectin-coated liposomes revealed that they were dispersed in a polymeric network of pectins. This could increase steric hindrance, which could be a beneficial way of protecting liposomes during transit in oral delivery. The pectin network can also help regulate the release of the active ingredient and may thus lead to improved uptake or absorption kinetics. Therefore, further characterization of the pectin-liposome constructs could enhance our understanding of the dynamics of pectin-coated liposomes, potentially leading to optimized formulations for various drug delivery applications.

## Data Availability

The datasets generated during the current study are available from the corresponding author upon reasonable request. A.L., Q.L., M.D. and S.T.S can verify the accuracy of the raw data for the study.

## References

[1] Fu Y, Saraswat A, Vartak R, Patki M, Patel K. Chapter 4 - Liposomal formulation: opportunities, challenges, and industrial applicability. In: Micro and Nano Technologies. Mehra NK, Srivastava S, Madan J, Kumar P, Singh BTMN., Eds. Elsevier. 2022, pp. 79–102. 10.1016/B978-0-323-85041-4.00021-4.

[2] Sercombe L, Veerati T, Moheimani F, Wu SY, Sood AK, Hua S. Advances and Challenges of Liposome Assisted Drug Delivery. Front Pharmacol. 2015;6:286. 10.3389/fphar.2015.00286. 26648870 10.3389/fphar.2015.00286 PMC4664963

[3] De Leo V, Milano F, Agostiano A, Catucci L. Recent Advancements in Polymer/Liposome Assembly for Drug Delivery: From Surface Modifications to Hybrid Vesicles. Polymers. 2021;13(7). 10.3390/polym13071027. 10.3390/polym13071027 PMC8037206 33810273

[4] Klemetsrud T, Kjøniksen A-L, Hiorth M, Jacobsen J, Smistad G. Polymer coated liposomes for use in the oral cavity – a study of the in vitro toxicity, effect on cell permeability and interaction with mucin. J Liposome Res. 2018;28(1):62–73. 10.1080/08982104.2016.1255640. 27809639 10.1080/08982104.2016.1255640

[5] Hou X, Zaks T, Langer R, Dong Y. Lipid nanoparticles for mRNA delivery. Nat Rev Mater. 2021;6(12):1078–94. 10.1038/s41578-021-00358-0. 34394960 10.1038/s41578-021-00358-0 PMC8353930

[6] Sahatsapan N, et al. Feasibility of mucoadhesive chitosan maleimide-coated liposomes for improved buccal delivery of a protein drug. J Drug Deliv Sci Technol. 2022;69:103173. 10.1016/j.jddst.2022.103173.

[7] C. Mellinas, M. Ramos, A. Jiménez, and M. C. Garrigós, “Recent Trends in the Use of Pectin from Agro-Waste Residues as a Natural-Based Biopolymer for Food Packaging Applications. Materials. 2020;3(3). 10.3390/ma13030673. 10.3390/ma13030673 PMC7042806 32028627

[8] Espitia PJP, Du WX, de JesúsAvena-Bustillos R, Soares NDFF, McHugh TH. Edible films from pectin: Physical-mechanical and antimicrobial properties - A review. Food Hydrocoll. 2014;35:287–96. 10.1016/j.foodhyd.2013.06.005.

[9] Freitas CM, Coimbra JS, Souza VG, Sousa RC. Structure and Applications of Pectin in Food, Biomedical, and Pharmaceutical Industry: A Review. Coatings. 2021;11(8). 10.3390/coatings11080922.

[10] Mohnen D. Pectin structure and biosynthesis. Curr Opin Plant Biol. 2008;11(3):266–77. 10.1016/j.pbi.2008.03.006. 18486536 10.1016/j.pbi.2008.03.006

[11] Noreen A, et al. Pectins functionalized biomaterials; a new viable approach for biomedical applications: A review. Int J Biol Macromol. 2017;101:254–72. 10.1016/j.ijbiomac.2017.03.029. 28300586 10.1016/j.ijbiomac.2017.03.029

[12] Kastner H, Einhorn-Stoll U, Drusch S. Structure formation in sugar containing pectin gels - Influence of gel composition and cooling rate on the gelation of non-amidated and amidated low-methoxylated pectin. Food Hydrocoll. 2017;73:13–20. 10.1016/j.foodhyd.2017.06.023. 10.1016/j.foodchem.2013.06.127 24099540

[13] Klemetsrud T, Jonassen H, Hiorth M, Kjøniksen A-L, Smistad G. Studies on pectin-coated liposomes and their interaction with mucin. Colloids Surf B Biointerfaces. 2013;103:158–65. 10.1016/j.colsurfb.2012.10.012. 23201733 10.1016/j.colsurfb.2012.10.012

[14] Thirawong N, Thongborisute J, Takeuchi H, Sriamornsak P. Improved intestinal absorption of calcitonin by mucoadhesive delivery of novel pectin–liposome nanocomplexes. J Control Release. 2008;125(3):236–45. 10.1016/j.jconrel.2007.10.023. 18082282 10.1016/j.jconrel.2007.10.023

[15] Wang Y, et al. Fabricating pectin and chitosan double layer coated liposomes to improve physicochemical stability of beta-carotene and alter its gastrointestinal fate. Int J Biol Macromol. 2023;247:125780. 10.1016/j.ijbiomac.2023.125780. 37433420 10.1016/j.ijbiomac.2023.125780

[16] Shende P, Patil A, Prabhakar B. Layer-by-layer technique for enhancing physicochemical properties of actives. J Drug Deliv Sci Technol. 2020;56:101519. 10.1016/j.jddst.2020.101519.

[17] Weber C, et al. Functionalization of Liposomes with Hydrophilic Polymers Results in Macrophage Uptake Independent of the Protein Corona. Biomacromol. 2019;20(8):2989–99. 10.1021/acs.biomac.9b00539. 10.1021/acs.biomac.9b00539 PMC6750830 31268685

[18] Nguyen TX, Huang L, Gauthier M, Yang G, Wang Q. Recent Advances in Liposome Surface Modification for Oral Drug Delivery. Nanomedicine. 2016;11(9):1169–85. 10.2217/nnm.16.9. 27074098 10.2217/nnm.16.9

[19] Awasthi AK, et al. Polydopamine-on-liposomes: stable nanoformulations{,} uniform coatings and superior antifouling performance. Nanoscale. 2020;12(8):5021–30. 10.1039/C9NR07770G. 32065189 10.1039/c9nr07770g

[20] Damiati S, Kompella UB, Damiati SA, Kodzius R. Microfluidic Devices for Drug Delivery Systems and Drug Screening. Genes. 2018;9(2). 10.3390/genes9020103. 10.3390/genes9020103 PMC5852599 29462948

[21] Lo CT, Jahn A, Locascio LE, Vreeland WN. Controlled self-assembly of monodisperse niosomes by microfluidic hydrodynamic focusing. Langmuir. 2010;26(11):8559–66. 10.1021/la904616s. 20146467 10.1021/la904616s

[22] Nordly P, et al. Incorporation of a synthetic mycobacterial monomycoloyl glycerol analogue stabilizes dimethyldioctadecylammonium liposomes and potentiates their adjuvant effect in vivo. Eur J Pharm Biopharm. 2011;77(1):89–98. 10.1016/J.EJPB.2010.10.001. 20940050 10.1016/j.ejpb.2010.10.001

[23] Webb C et al. The Impact of Solvent Selection: Strategies to Guide the Manufacturing of Liposomes Using Microfluidics. Pharmaceutics. 2019;11(12). 10.3390/pharmaceutics11120653. 10.3390/pharmaceutics11120653 PMC6955969 31817217

[24] Lombardo D, Kiselev MA. Methods of Liposomes Preparation: Formation and Control Factors of Versatile Nanocarriers for Biomedical and Nanomedicine Application. Pharmaceutics. 2022;14(3). 10.3390/pharmaceutics14030543. 10.3390/pharmaceutics14030543 PMC8955843 35335920

[25] Yu M, et al. Temperature- and rigidity-mediated rapid transport of lipid nanovesicles in hydrogels. Proc Natl Acad Sci U S A. 2019;116(12):5362–9. 10.1073/PNAS.1818924116/SUPPL_FILE/PNAS.1818924116.SM06.AVI. 30837316 10.1073/pnas.1818924116 PMC6431219

[26] Benne N, et al. Atomic force microscopy measurements of anionic liposomes reveal the effect of liposomal rigidity on antigen-specific regulatory T cell responses. J Control Release. 2020;318:246–55. 10.1016/J.JCONREL.2019.12.003. 31812539 10.1016/j.jconrel.2019.12.003

[27] Nguyen S, Alund SJ, Hiorth M, Kjøniksen A-L, Smistad G. Studies on pectin coating of liposomes for drug delivery. Colloids Surf B Biointerfaces. 2011;88(2):664–73. 10.1016/j.colsurfb.2011.07.058. 21862293 10.1016/j.colsurfb.2011.07.058

[28] Xie F, et al. Pectins of different resources influences cold storage properties of corn starch gels: Structure-property relationships. Food Hydrocoll. 2022;124:107287. 10.1016/j.foodhyd.2021.107287.

[29] Bonavita A, Carratore V, Ciardiello MA, Giovane A, Servillo L, D’Avino R. Influence of pH on the Structure and Function of Kiwi Pectin Methylesterase Inhibitor. J Agric Food Chem. 2016;64(29):5866–76. 10.1021/acs.jafc.6b01718. 27335009 10.1021/acs.jafc.6b01718

